# Multi-consensus formation control by artificial potential field based on velocity threshold

**DOI:** 10.3389/fnins.2024.1367248

**Published:** 2024-03-25

**Authors:** Xiaofei Chang, Jiayue Jiao, Yuenan Li, Bei Hong

**Affiliations:** ^1^Northwestern Polytechnical University, Xi’an, China; ^2^CAMA, Luoyang, China; ^3^Beijing Institute of Astronautical Systems Engineering, Beijing, China

**Keywords:** artificial potential field, formation control, multi-consensus, velocity threshold, swarm motion potential function

## Abstract

This study proposes a multi-consensus formation control algorithm by artificial potential field (APF) method based on velocity threshold. The algorithm improves the multi-consensus technique. This algorithm can split a group of agents into multiple agent groups. Note that the algorithm can easily complete the queue transformation as long as the entire proxy group is connected initially and no specific edges need to be removed. Furthermore, collision avoidance and maintenance of existing communication connectivity should be considered during the movement of all agents. Therefore, we design a new swarm motion potential function. The stability of multi-consensus formation control has proven to be effective in avoiding collisions, maintaining connectivity, and generating formations. The final numerical simulation results show the role of the controller we designed.

## 1 Introduction

With its great application potential, multi-agent systems (MAS) have experienced rapid development in both theoretical research and engineering applications in recent years. In terms of information acquisition, fault tolerance, and completion of complex tasks, MAS has incomparable advantages over a single agent (Sui et al., 2020a,b). Currently, MAS has found extensive research applications in precise mapping, environmental monitoring, and military domains, among others.

**Figure 1:**
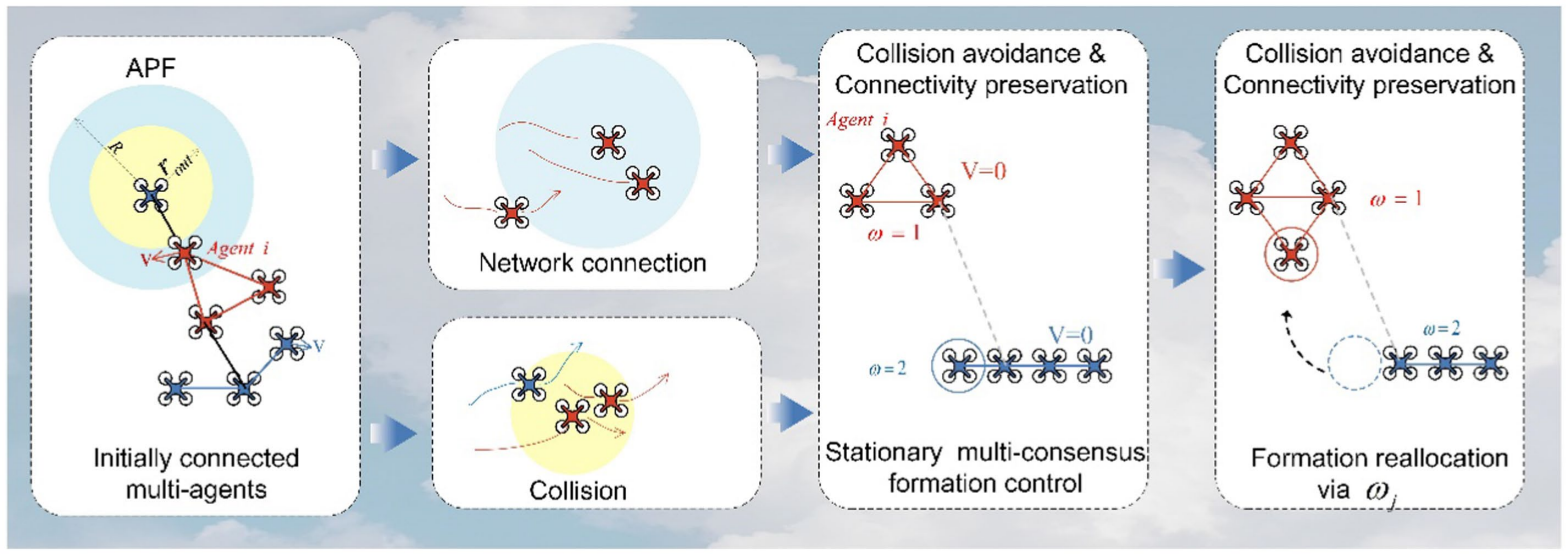
Concept of multi-consensus formation control.

Formation control is a critical research area in the field of MAS, involving collaborative behaviors among multiple agents to achieve specific tasks or objectives. The primary methods for formation control include leader–follower methods, virtual structure methods, APF methods, and distributed control methods (Wu and Li, 2023), among others. Leader–follower methods (Yu and Chen, 2021) are one of the most common strategies in formation control. In this approach, one or more agents are designated as leaders, and their actions dictate the collective behavior of the entire formation. The remaining agents act as followers, adjusting their positions or velocities to maintain specific relative positions with respect to the leaders. This method is often used in hierarchical tasks where leaders provide guidance and objectives for the formation. Virtual structure methods introduce virtual connections or structures, such as spring-mass systems, to achieve formation control. In this approach, each agent is influenced by virtual connections with adjacent agents. These virtual connections are defined using models like spring systems or other forms of interactions to maintain the desired formation shape. Virtual structure methods (Ren and Beard, 2005) are typically employed in tasks that require maintaining specific geometric configurations, such as multi-robot formations or aerial vehicle formations. APF methods emulate the concepts of physical fields and forces in the natural world to guide agents toward specific goals or shapes. In this method, each agent is influenced by a potential field that directs them to avoid obstacles and collaborate with other agents. APF methods (Li et al., 2022) are widely used in robot navigation and autonomous flight due to their adaptability and real-time characteristics. Distributed control (Wang et al., 2019) methods emphasize local communication and decision-making among agents to coordinate their behaviors without the need for a central coordinator. This approach is particularly useful in large-scale MAS, allowing agents to adapt in real time to changing environments and tasks. Distributed control methods include consensus-based methods, model predictive control, and reinforcement learning, among others. The book “Graph Theoretic Methods in Multiagent Networks” (Mehran and Egerstedt, 2010) provides an overview of the application of graph theory methods in multi-agent networks. It explores the applications of distributed control methods in MAS. The choice of formation control method typically depends on the nature of the task, interactions between agents, and environmental conditions. However, traditional control methods are only suitable for scenarios where multiple agents form a single coherent MAS. In practice, situations can be more complex, requiring different formations to accomplish multiple objectives. Therefore, MAS needs to establish multi-consensus formation control as depicted in Figure 1.

In the realm of formation control, achieving consensus among multiple agents is a fundamental and intricate challenge. Beyond traditional consensus, where agents aim to converge to a single common value, multi-consensus adds a layer of complexity by targeting agreement on multiple quantities of common interest. This nuanced concept opens up new avenues for research and applications, addressing scenarios where diverse objectives must be met within a multi-agent system (Han et al., 2016). Ren and Beard (2005) made significant contributions to the field by analyzing the convergence of high-order MAS operating under consensus algorithms. Their work not only delved into the theoretical underpinnings of convergence but also provided necessary and sufficient conditions for achieving it. Their research is instrumental in understanding the theoretical foundations of multi-consensus. Guo et al. (2014) introduced an innovative distributed event-triggered transmission strategy. This strategy facilitates MAS in achieving multi-consensus by allowing agents to converge to different values at different stages of the interaction. This adaptive approach recognizes that within complex tasks, not all agents need to reach the same consensus simultaneously, but rather, multiple consensuses can evolve iteratively, aligning with the dynamics of the task. Zhu et al. (2015) proposed a segmental unmanned aerial vehicle (UAV) formation control strategy founded on the principle of information consistency. They not only presented the concept but also conducted comprehensive simulations to validate the rationality and effectiveness of the strategy. Such real-world application of multi-consensus strategies is crucial for confirming their practical utility.

In addition, it is crucial to underscore the paramount significance of collision avoidance and the maintenance of connectivity in the realm of multi-subgroup formation control. These two aspects remain pivotal challenges that MAS grapple with, particularly during the intricate process of switching network topologies (Dong et al., 2021). Current research indicates that MAS network topologies are often artificially designed, introducing a level of uncertainty and sub-optimality. This uncertainty can pose hindrances to the stable preservation of formations under multi-agent control, necessitating innovative solutions. To address the trajectory tracking problem, noteworthy efforts have been made by researchers. For instance, Mondal et al. have devised a pioneering multi-agent formation control approach that not only steers clear of collisions but also meticulously upholds connectivity within the system (Mondal et al., 2017). On a similar note, Liu et al. (2019) have proposed a distinctive MAS formation generation strategy hinged on a network topology that employs an event-triggered mechanism. Although this method showcases promise, it does require the establishment of internal topology connections within sub-formations. These endeavors mark significant advancements in the field and offer potential solutions to these persistent challenges.

Based on the aforementioned analysis, this study conducts research on the multi-agent formation control problem and presents an APF method suitable for multi-subgroup formations based on velocity thresholds. This approach constructs communication topologies, allowing agents to maintain predefined formations effectively. The main contributions are summarized as follows:

- Based on an enhancement of established multi-consensus algorithms, we introduce a novel decentralized multi-agent consensus formation control algorithm. This innovation involves the decentralization of a set of MAS into multiple groups, eliminating the requirement for subgroup network conditions;
- In our quest to achieve precise trajectory tracking and ensure sustained connectivity in the domain of multi-subgroup formation control, we have developed a sophisticated group motion potential energy function inspired by the principles of artificial potential fields. This innovative approach serves as a guiding force, orchestrating the coordinated movement of the agents in complex environments, thereby enabling seamless trajectory tracking and effective maintenance of connectivity;
- Furthermore, our comprehensive strategy involves the implementation of tailored velocity thresholds for each individual agent. These specific thresholds are meticulously designed to optimize the stability and safety of the system while enhancing the ability of the agents to navigate within the formation. This dual-pronged approach not only advances the field of MAS but also opens new avenues for addressing collaborative control challenges across various domains.

## 2 Preliminaries

### 2.1 Graph theory

In this paper, the weighted undirected graph is used to describe the communication relationship between the agents in the system (Luo and Cao, 2014).

First, in this paper, the undirected graph is defined as G . We define the set of points of the undirected graph as $V=\left\{1,2,\ldots ,n\right\}$ and the set of edges as $Ε=\left\{e_{ij}|i,j\in V,i\neq j\right\}$ . Then, the adjacency matrix is defined as $A\in R^{n\times n}$ . n denotes the number of agents in the system. The elements of the adjacency matrix are $a_{ij}$ , indicating the adjacency of the i -th vertex at the j -th vertex. Agent i and agent j are concatenated when $e_{ij}\in E$ and $a_{ij}=1$ , but not vice versa. Furthermore, when $i=j,a_{ij}=0$ . In addition, if agent i is connected to agent j , information about each other can be obtained when $e_{ij}$ exists between agents. The connection set for agent i is defined as $N_{i}=\left\{j|e_{ij}\right\}$ . The degree matrix of the graph is defined as D: $=\;\mathrm{diag}\left\{d_{1},d_{2},\ldots ,d_{n}\right\}$ where $d_{i}=\overset{n}{\underset{j=1}{\sum }}a_{ij}$ . The Laplacian matrix L of the unweighted undirected graph can be expressed as follows:


(1) L=D−A

When the communication is restricted, the communication strength between agent i and agent j is affected by the relative distance. Considering the above situation, the element $a_{ij}$ in the adjacency matrix A needs to be modified. The modified matrix elements are values on a continuous space, and $a_{ij}$ can be expressed as follows:


(2) $${\bar{a}}_{ij}\left(∥x_{ij}\left(t\right)∥\right)=\{\begin{array}{c} 1\\ \frac{1}{2}\left[1+\cos(\pi \frac{\frac{∥x_{ij}\left(t\right)∥}{R}-\tau }{1-\tau }\right]\\ 0 \end{array}\;\begin{array}{c} \;∥x_{ij}\left(t\right)∥\;\in [0,\tau R)\\ ∥\;x_{ij}\left(t\right)∥\;\in [\tau R,R)\\ \;∥x_{ij}\left(t\right)∥\;\in [R,\inf) \end{array}$$

where we define τ as the communication-limited decay rate and R as the farthest communication boundary. $x_{ij}\left(t\right)$ is denoted as the distance between agent i and agent j at moment $t,x_{ij}\left(t\right)=x_{i}\left(t\right)-$ $x_{j}\left(t\right).∥\cdot ∥$ is the 2-norm. The model is referenced from Fang et al. (2016). $\bar{A}$ can be defined as a n×n matrix whose $\left(i,j\right)$ element is $a_{ij},\bar{D}:\;=\;\mathrm{diag}\left\{{\bar{d}}_{1},\;{\bar{d}}_{2},\ldots ,\;{\bar{d}}_{n}\right\}$ , and ${\bar{d}}_{i}=\overset{n}{\underset{j=1}{\sum }}{\bar{d}}_{ij}$ . The weighted Laplacian matrix is defined as follows:


(3) $$\bar{L}=\bar{D}-\bar{A}$$

It is worth noting that each element in $\bar{L}$ is a time-varying continuous function between related agents. Furthermore, the elements of L are closely related to the undirected time-varying graph G . $a_{ij}$ may jump when G changes. Unless otherwise stated, all graphs in this paper are undirected graphs.

### 2.2 Multi-consensus

We describe the dynamics of a second-order multi-agent system with n agents as follows:

(4) $$\left\{ \begin{array}{l} {\dot{x}}_{i}\left(t\right)=v_{i}\left(t\right),\\ {\dot{v}}_{i}\left(t\right)=u_{i}\left(t\right),\forall i\in V \end{array}\right.$$

where $u_{i}\left(t\right)$ is the control method to be designed in this study. It is obvious that $x_{i}\left(t\right)\in R$ represents the position of agent i and $v_{i}\left(t\right)\in R$ represents the velocity of agent i . Additionally, all assumed states in this study are one-dimensional. The Kronecker (Choi et al., 2020) product can be used if it is necessary to extend the methods of this study and the states such as position to multiple dimensions.

In a second-order (Pan and Nian, 2014) multi-intelligence system (Eq. 4) with *n* agents, multi-agent is considered to have reached consensus if they achieve a smooth consistency. Smooth consistency between agents i and j is defined as follows (Han et al., 2016):


(5) $$\left\{\begin{array}{l} \underset{t\to \infty }{\lim}∥x_{i}\left(t\right)-x_{j}\left(t\right)∥=0\\ \underset{t\to \infty }{\lim}∥v_{i}\left(t\right)=\underset{t\to \infty }{\lim}v_{j}\left(t\right)=0 \end{array}\right.$$

The consensus of agents implies that their states reach an agreement. By achieving identical states, the MAS can accomplish collective behavior (Gulzar et al., 2018).

**Lemma 1 [Laplacian matrix (****Han et al., 2016****)].** *We define a Laplacian matrix* $\hat{L}$ *under multiple consensus:*


(6) $$\hat{L}=\left[\begin{array}{cccc} L_{11} & \frac{\omega_{1}}{\omega_{2}}L_{12} & \cdots & \frac{\omega_{1}}{\omega_{n}}L_{1n}\\ \frac{\omega_{2}}{\omega_{1}}L_{21} & L_{22} & \cdots & \frac{\omega_{2}}{\omega_{n}}L_{2n}\\ \vdots & \vdots & \ddots & \vdots \\ \frac{\omega_{n}}{\omega_{1}}L_{n1} & \frac{\omega_{n}}{\omega_{2}}L_{n2} & \cdots & L_{nn} \end{array}\right]$$

*where* $\omega_{i}$ *means the intelligence degree of the agent* $i.L_{ij}$ *indicates the* $\left(i\text{,}j\right)$ *element of the Laplacian matrix* L .

In an MAS, agents, i and j can achieve a common consensus if agent i and j have the same degree of intelligence, i.e., the agents i and j satisfy Eq. (5) if $\omega_{i}=\omega_{j}$ . In another sentence, with multiple different degrees of intelligence, the MAS can achieve multiple consensus. Accordingly, the MAS can form different numbers of clusters through assigning the appropriate degree of intelligence. Compared to traditional consensus protocols that aggregate all agents to the same state, this study uses intelligence to achieve multiple consensuses in a more convenient way. More details can be found in Han et al. (2016).

### 2.3 Artificial potential field

The APF method is a classic control algorithm with universality. Various APF methods have been proposed for formation control (Yu and Chen, 2020). The algorithm regards the target and the obstacle as objects with attraction and repulsion to the agent, respectively, and the agent moves along the resultant force of attraction and repulsion.

**Figure 2:**
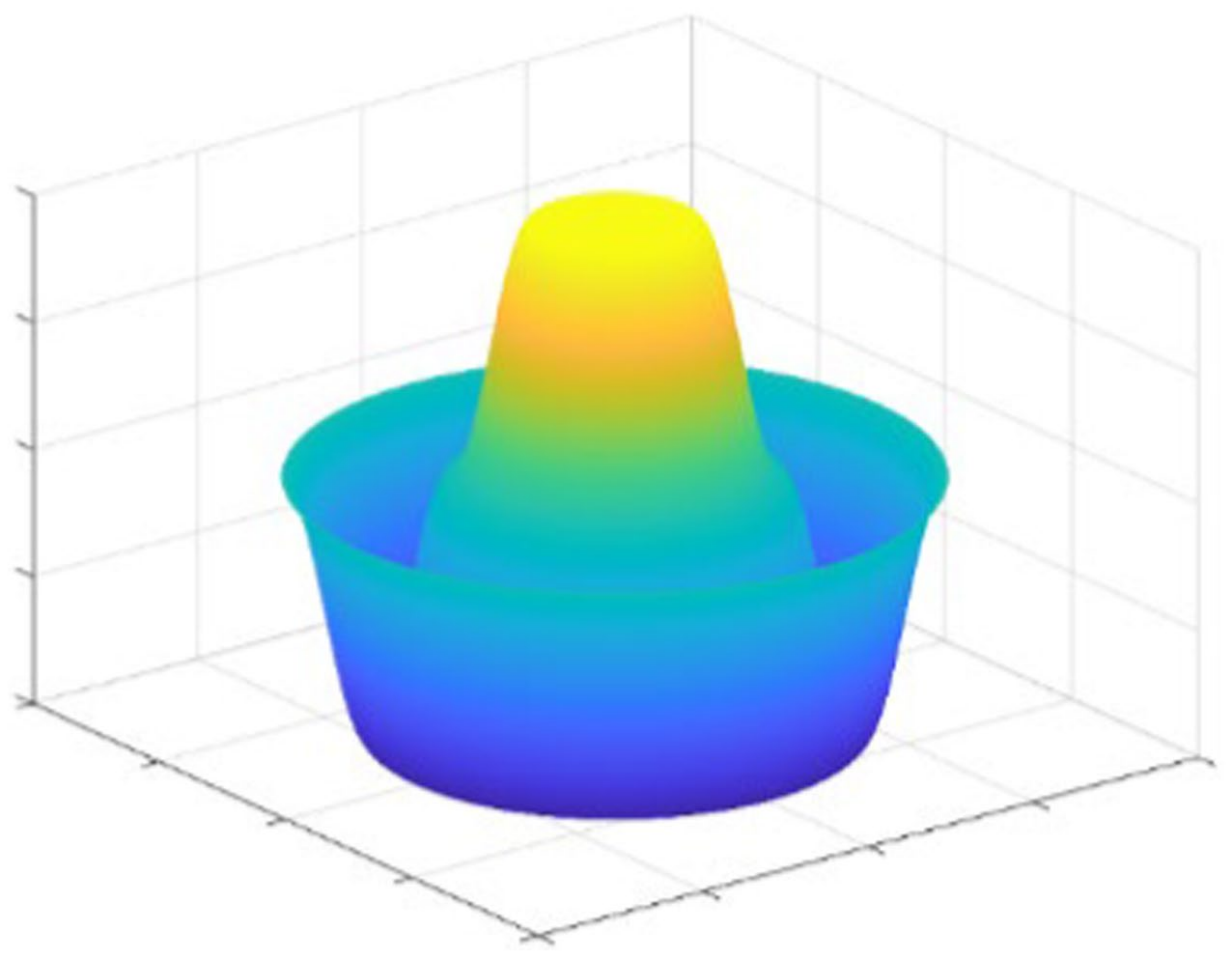
Example of the potential field.

The sticking point to the algorithm is to construct an artificial potential field. Suppose the agents move in a plane, we would get the potential field as shown in Figure 2. The peak in the figure is the potential field at the location of each agent. The potential field generates attractive forces at a distance from the agent before reaching the position specified in the formation, and the potential field can also generate repulsive forces at a closer distance from the agent.

### 2.4 Problem formation

In multi-subgroup decentralized control, agents need to consider avoiding collisions and maintaining connectivity.

To this end, we design a suitable artificial potential energy field algorithm. The group motion potential functions $V\left(t\right)$ we designed include collision avoidance potential function $G_{1}\left(t\right)$ and connectivity preserving potential function $\;G_{2}\left(t\right)$ . Due to the potential energy field having a certain range of action, the collision avoidance potential function satisfies $G_{1}\left(t\right)<G_{1}^{\max}$ . Similarly, $G_{2}\left(t\right)<G_{2}^{\max}$ to maintain connectivity. To ensure that the designed formation control law works at all times, the designed group motion potential function should satisfy


(7) $$\{\begin{array}{l} V\left(t\right)\leq G_{1}^{\max}\\ V\left(t\right)\leq G_{2}^{\max} \end{array}$$

## 3 Controller design and stability analysis

This section proposes the multi-consensus control method, and we prove the stability of the control method theoretically using the Lyapunov function approach.

### 3.1 Obstacle avoidance strategy design

The safe distance from the obstacle is $r_{in}^{t}$ , and the working boundary is $r_{out}^{t}$ . In other words, the obstacle avoidance potential function takes effect when $r_{in}^{t}\leq x_{ij}\leq r_{out}^{t}$ . The obstacle avoidance boundary is smaller than the communication distance, so each agent can detect obstacles within the obstacle avoidance area.

Define the collision avoidance neighbor of agent i as $N_{i}^{q}$ , which can be denoted as


(8) $$N_{i}^{q}=\left\{j\in \left\{1\text{,}\cdots \text{,}n\right\}:\;\;∥x_{i}-x_{q}∥\;\;\leq r_{\text{out}}\right\}$$

where $x_{q}$ is the position of the obstacle.

The potential function is designed as follows:


(9) $$G_{1}\left(x_{ij}\right)=\{\begin{array}{cc} k_{1}\overset{r_{\mathrm{out}}}{\underset{x_{ij}}{\int }}g_{1}\left(s\right)ds, & x_{ij}\in \left[r_{\mathrm{in}},r_{\mathrm{out}}\right]\\ 0, & \;\mathrm{otherwise}\; \end{array}$$

where


(10) $$g_{1}\left(x_{ij}\right)=\frac{1}{2}\left[1+\cos\left(\pi \frac{x_{ij}-r_{in}}{r_{out}-r_{in}}\right)\right]$$

Then, the collision avoidance control for agent i at $x_{i}$ is defined as


(11) $$u_{i}^{q}=\underset{j\in N_{i}^{q}}{\sum }\nabla_{x_{i}}G_{1}\left(x_{ij}\right)$$

where $\nabla_{x_{i}}$ is the gradient along $x_{i}$ .

In the collision avoidance area, the relative distance between the two agents is less than $r_{out}$ . The smaller the relative distance, the greater the input of the control law. The MAS can comply with collision avoidance.

### 3.2 Connectivity preservation

This part is designed to keep the intelligent agent connection within a certain range R . When $r_{out}<x_{ij}\leq R$ , the formation maintenance function works. As communication between agents is limited, for agent i in the communication range, there is a set of connectable agent ensembles denoted as $N_{i}^{n}$ .


(12) $$N_{i}^{l}=\left\{j\in \left\{1\text{,}\cdots \text{,}n\right\}:i∼j\right\}$$

where i∼j indicates $\left(i\text{,}j\right)\in E$ or $\left(j\text{,}i\right)\in E$ , which means that communication is possible between agent i and agent j .

The connectivity-preserving potential function is designed as follows:


(13) $$G_{2}\left(x_{ij}\right)=\{\begin{array}{cc} k_{2}\overset{\rho_{ij}}{\underset{x}{\int }}g_{2}\left(s\right)ds, & x_{ij}\in \left(r_{out},R\right]\\ 0, & \;\mathrm{otherwise}\; \end{array}$$

where


(14) $$g_{2}\left(x_{ij}\right)=\{\begin{array}{cc} \frac{1}{2}\left[1+\cos\left(\pi \frac{x_{ij}-r_{\text{out}}}{d_{ij}-r_{\text{out}}}\right)\right], & x_{ij}\in \left(r_{\text{out}}\text{,}\rho_{ij}\right]\\ 1+\cos\left(\pi \frac{x_{ij}-R}{R-\rho_{ij}}\right), & x_{ij}\in \left(\rho_{ij}\text{,}R\right] \end{array}$$

where $\rho_{ij}=\rho_{i}-\rho_{j}$ refers to the required relative position. $\rho_{i}$ refers to the target state of the agent i when forming a formation shape.

Then, the connectivity preservation control input of agent i can be designed as


(15) $$u_{i}^{l}=\underset{j\in N_{i}^{l}}{\sum }\nabla_{x_{i}}G_{2}\left(x_{ij}\right)$$

In addition, we design the potential function $G_{3}\left(x_{ij}\right)=1$ when $x_{ij}>R$ . This design allows the connection to be broken between agents if necessary. Therefore, the network can be flexibly changed to achieve the desired formation.

Another point to note, the agent spacing of the expected formation should be set between on $\left[r_{out}\text{,}R\right]$ . In other words, the states of agent i and agent j need to satisfy the following inequation:


(16) $$0<r_{\text{out}}\leq \;\;∥\rho_{ij}∥\;\;\leq R,\forall i,j\in V,\omega_{i}=\omega_{j},\;\;i\neq j$$

### 3.3 Decentralized multi-consensus formation control method

For each sub-formation in the MAS system described in Eq. (4), the relative configurations between the agents of the formation subgroups designed in this study need to satisfy the following requirements:


(17) $$x_{ij}=\rho_{ij},\forall i,j\in V,i\neq j,\omega_{i}=\omega_{j}.$$

This study is designed with displacement-based formations. Displacement-based formation (Fang et al., 2016) is a different type of formation than distance-based formation that may flip or rotate a designated graph. The configuration of MAS is unique under this method.

To solve the multi-subgroup formation problem, we propose a multi-consensus formation algorithm. The key of this algorithm is to redistribute the formation of different subgroups by changing the intelligence degree $\omega_{i},\forall i\in V$ of the agent. The difference in $\omega_{i}$ also affects the different distances between subgroups (Oh et al., 2015).

During formation control, only the subgroup formation information including $\rho_{i}$ and $\omega_{i}$ needs to be specified and no other manual settings are required. In the initial state, there can be agents that are not connected to the subgroups in each subgroup formation, but the network of the MAS needs to be connected.

**Definition 1.** *As usual, the condition for formation of a formation is that the state between agents satisfies (**Eq. 17**). Referring to the quasi* α *-lattice concept in (*Choi et al., *2019**), this study considers the effect of error* $\left(\delta >0\right)$ *in the formation of the formation. The following inequality is generated.*


(18) $$\rho_{ij}-\delta \leq x_{ij}\leq \rho_{ij}+\delta ,\forall i,j\in V,i\neq j\;\text{and}\;\omega_{i}=\omega_{j}$$

The above inequality shows that the formation formed by MAS is a formation with a certain error influenced by δ .


(19) $$\delta =\underset{\omega_{i}=\omega_{j}}{\max}∥x_{ij}-\rho_{ij}∥$$

**Figure 3:**
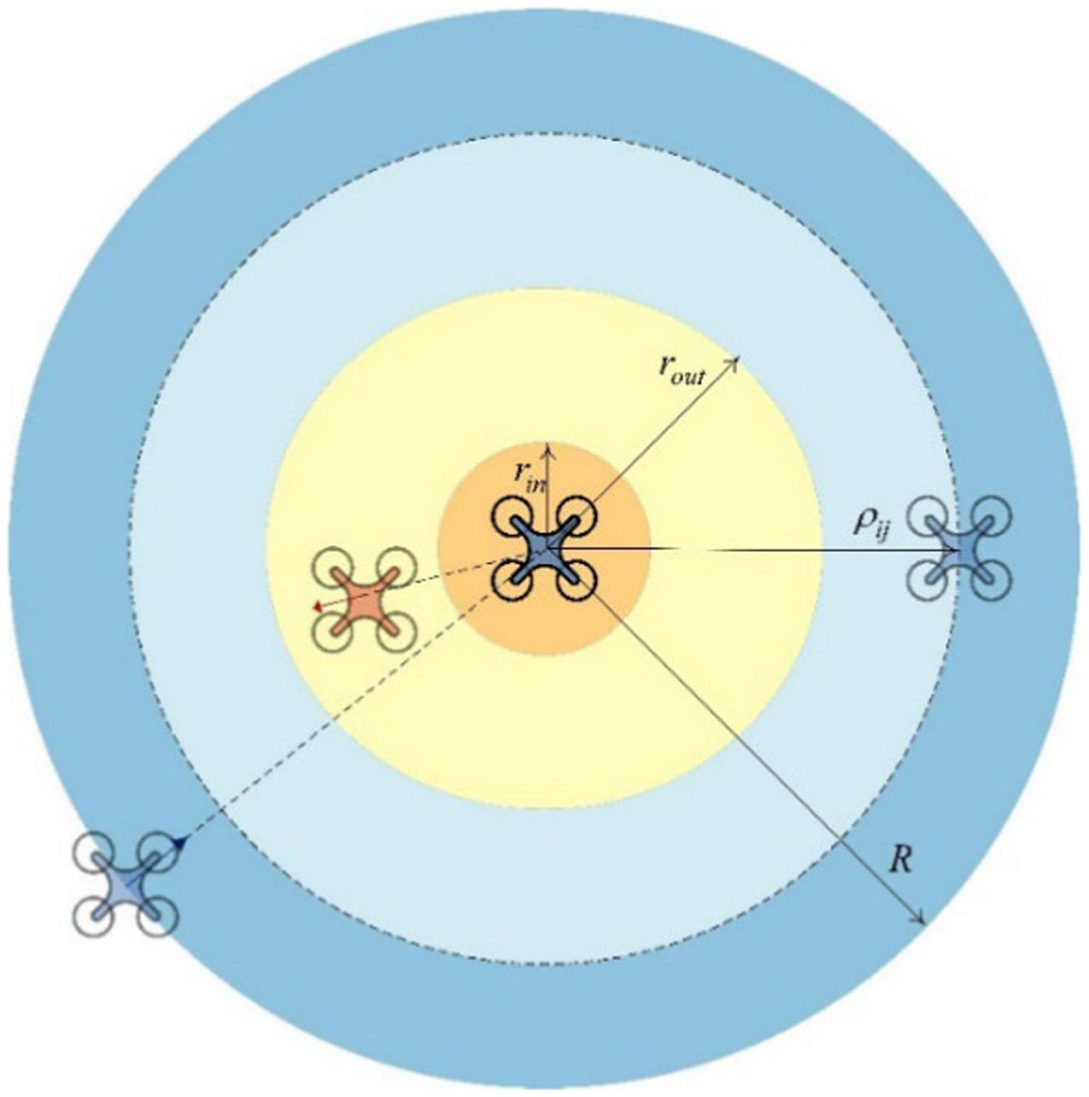
APF function area between agents.

In this study, we propose decentralized multi-consensus control methods with collision avoidance and connectivity preservation:


(20) $$\begin{array}{rr} u_{i}= & -\omega_{i}^{2}\underset{j\in N_{i}^{t}}{\sum }\nabla_{x_{i}}G_{1}\left(x_{ij}\right)-\omega_{i}^{2}\underset{j\in N_{i}^{l}}{\sum }\nabla_{x_{i}}G_{2}\left(x_{ij}\right)-\\ & \alpha \underset{j\in N_{i}}{\sum }S\left(\varepsilon_{i}^{\rho },\varepsilon_{j}^{\rho },v_{m}\right)-\beta v_{i} \end{array}$$

where


(21) $$S\left(\varepsilon_{i}^{\rho },\varepsilon_{j}^{\rho },v_{m}\right)≜\left\{\begin{array}{c} \frac{v_{m}}{\left|\varepsilon_{i}^{\rho }-\frac{\omega_{i}}{\omega_{j}}\varepsilon_{j}^{\rho }\right|}\left(\varepsilon_{i}^{\rho }-\frac{\omega_{i}}{\omega_{j}}\varepsilon_{j}^{\rho }\right)\\ \varepsilon_{i}^{\rho }-\frac{\omega_{i}}{\omega_{j}}\varepsilon_{j}^{\rho } \end{array}\right.\begin{array}{c} \;\left|\varepsilon_{i}^{\rho }-\frac{\omega_{i}}{\omega_{j}}\varepsilon_{j}^{\rho }\right|>v_{m}\\ \;\left|\varepsilon_{i}^{\rho }-\frac{\omega_{i}}{\omega_{j}}\varepsilon_{j}^{\rho }\right|>v_{m} \end{array}$$

where $v_{m}$ is the maximum safe speed $.\varepsilon_{k}^{p}=x_{k}-\rho_{k},k=$ 1,2,…,n , and α,β>0 . When the subgroup reaches the target, $v_{i}=0$ . We can also obtain the APF function between $\left(r_{in}\text{,}R\right)$ of agent l in the group, as shown in Figure 3.

### 3.4 Stability analysis

This part is about the proof of system stability. First, we define the Lyapunov function (Xin et al., 2023).

**Definition 2.** *(*Choi et al., *2019**) For the input (**Eq. 20**) proposed in this study, the Lyapunov function is defined as follows:*


(22) $$\begin{array}{rr} V= & \;\overset{N}{\underset{i=1}{\sum }}\left\{\underset{j\in N_{i}^{q}}{\sum }G_{1}\left(x_{ij}\right)+\underset{j\in N_{i}^{l}}{\sum }G_{2}\left(x_{ij}\right)+\right.\\ & \left.{\left(\frac{1}{\omega_{i}}\right)}^{2}\alpha \overset{t}{\underset{0}{\int }}S{\left(\varepsilon_{i}^{\rho },\varepsilon_{j}^{\rho },v_{m}\right)}^{T}d\varepsilon_{i}^{\rho }+\overset{n}{\underset{i=1}{\sum }}{\hat{v}}_{i}^{2}\right\} \end{array}$$

*where* ${\hat{v}}_{i}$ *is* $\frac{v_{i}}{\omega_{i}}$ .

We want to prove the stability proof of the system needs to consider two cases. First, we demonstrate the stability of the MAS network when the connection is fixed. Then, this study proves the stability when the topology is dynamically changed.

**Theorem 1.** *(*Xin et al., *2023**) When the control input is multi-consensus control method (**Eq. 20**), the MAS with a fixed network topology of static and dynamics of* *Eq. (4)* *can achieve any number (* ≤n *) of formation control. For the target formation, there are the following conditions to be satisfied:*

-
$\omega_{i}\neq 0,\forall i\in V$

-
$\text{V}\left(0\right)$ *is bounded*
- *The displacement between agents needs to satisfy* *Eq. (16)*

***Proof.*** To prove the stability of the Lyapunov function V , it is necessary to derive it. We first compute the $G_{1}$ part of the Lyapunov function.


(23) $$\underset{j\in N_{i}^{q}}{\sum }{\dot{G}}_{1}\left(x_{ij}\right)={\left({\dot{x}}_{i}\right)}^{T}\underset{j\in N_{i}^{q}}{\sum }\nabla_{x_{i}}G_{1}\left(x_{ij}\right)$$

where ${\left({\dot{x}}_{i}\right)}^{T}={\left(v_{i}\right)}^{T}$ and


(24) $$\overset{t}{\underset{0}{\int }}S{\left(\varepsilon_{i}^{\rho },\varepsilon_{j}^{\rho },v_{m}\right)}^{T}d\varepsilon_{i}^{\rho }=\overset{t}{\underset{0}{\int }}S{\left(\varepsilon_{i}^{\rho },\varepsilon_{j}^{\rho },v_{m}\right)}^{T}v_{i}\;d\tau$$

According to the above equation, the derivative of V can be transformed into


(25) $$\dot{V}=\overset{N}{\underset{i=1}{\sum }}\left\{\begin{array}{l} {\left({\dot{x}}_{i}\right)}^{T}\underset{j\in N_{i}^{q}}{\sum }\nabla_{x_{i}}G_{1}\left(x_{ij}\right)+{\left({\dot{x}}_{i}\right)}^{T}\underset{j\in N_{i}^{q}}{\sum }\nabla_{x_{i}}G_{2}\left(x_{ij}\right)\\ +\alpha {\left(\frac{1}{\omega_{i}}\right)}^{2}S{\left(\varepsilon_{i}^{\rho },\varepsilon_{j}^{\rho },v_{m}\right)}^{T}v_{i}+{\hat{v}}_{i}\dot{{\hat{v}}_{i}} \end{array}\right\}$$

in Eq. (25), the following equation exists


(26) $$\overset{n}{\underset{i=1}{\sum }}{\hat{v}}_{i}\dot{{\hat{v}}_{i}}=\overset{n}{\underset{i=1}{\sum }}\frac{{\hat{v}}_{i}u_{i}}{\omega_{i}}$$

where $\hat{u}={\left[\frac{u_{1}}{\omega_{1}},\frac{u_{2}}{\omega_{2}},\ldots ,\frac{u_{n}}{\omega_{n}}\right]}^{T}$ , and $\dot{V}$ can be rewritten as


(27) $$\dot{V}=\overset{N}{\underset{i=1}{\sum }}\left\{\begin{array}{l} {\left(v_{i}\right)}^{T}\underset{j\in N_{i}^{q}}{\sum }\nabla_{x_{i}}G_{1}\left(x_{ij}\right)+{\left(v_{i}\right)}^{T}\underset{j\in N_{i}^{l}}{\sum }\nabla_{x_{i}}G_{2}\left(x_{ij}\right)\\ +\alpha {\left(\frac{1}{\omega_{i}}\right)}^{2}S{\left(\varepsilon_{i}^{\rho },\varepsilon_{j}^{\rho },v_{m}\right)}^{T}v_{i}+\frac{{\hat{v}}_{i}u_{i}}{\omega_{i}} \end{array}\right\}$$

here, $v={\left[v_{1}\text{,}v_{2}\text{,}\ldots \text{,}v_{n}\right]}^{T}$ . Let $\Lambda ≜diag\left[\omega_{1}\text{,}\omega_{2}\text{,}\ldots \text{,}\omega_{n}\right]$ , and we define


$$G_{1}^{*}=\left[\underset{j\in N_{i}^{q}}{\sum }\nabla_{x_{1}}G_{1}\left(x_{1j}\right),\underset{j\in N_{i}^{q}}{\sum }\nabla_{x_{2}}G_{1}\left(x_{2j}\right),\cdots \right.,{\left.\underset{j\in N_{i}^{q}}{\sum }\nabla_{x_{n}}G_{1}\left(x_{nj}\right)\right]}^{T}$$

The definition of $G_{2}^{*}$ is the same as that of $G_{1}^{+}$ , and


$$S^{*}=\left[s\left(x_{1}^{\rho }\text{,}x_{j}^{\rho }\text{,}v_{m}\right)\text{,}s\left(x_{2}^{\rho }\text{,}u_{j}^{\rho }\text{,}v_{m}\right)\text{,}\cdots \text{,}s\left(u_{m}^{\rho }\text{,}\varepsilon_{j}^{p}\text{,}v_{m}\right)\right]$$

according to the Eq. (20), then


(28) $$\dot{V}=v^{T}G_{1}^{*}+v^{T}G_{2}^{*}+\alpha \Lambda^{-2}v^{T}S^{*}+{\hat{v}}^{T}\hat{u}$$

one can easily notice $\hat{u}=\Lambda^{-1}u,\;\hat{v}=\Lambda^{-1}v$ and u can be written as


(29) $$u=-\Lambda^{2}G_{1}^{*}-\Lambda^{2}G_{2}^{*}-\alpha S^{*}-\beta v$$

Bringing the multi-consensus control law n into the derivative of the Lyapunov function, we get


$$\dot{V}=v^{T}G_{1}^{*}+\nu^{T}G_{2}^{*}+\alpha \Lambda^{-2}v^{T}S^{*}+{\hat{v}}^{T}\Lambda^{-1}\left(-\Lambda^{2}G_{1}^{*}-\Lambda^{2}G_{2}^{*}-\alpha S^{*}-\beta v\right)$$

$$=\alpha \Lambda^{-2}v^{T}S^{*}-\alpha {\hat{v}}^{T}\Lambda^{-1}S^{*}-\beta {\hat{v}}^{T}\hat{v}$$

(30) $$=-\beta {\hat{v}}^{T}\hat{v}\leq 0\;$$

The Lyapunov’s stability determination requires two proofs. The first point is that V is a semi-positive definite and the second point is that the derivative of V is negative semi-definite. According to Eq. (30), the second point can be obtained. In this study, the initial value $V_{0}$ of the Lyapunov function is constant and V is positive and constant at all times, so it is always positive and finite.


(31) $$V\left(t\right)\leq V\left(0\right)=\Theta \ll \infty ,\forall t\geq 0$$

The Lyapunov function needs to satisfy two threshold constraints to achieve collision avoidance and connectivity preservation.


(32) $$\{\begin{array}{l} V\left(t\right)\leq G_{1}\left(r_{in}\right)\\ V\left(t\right)\leq G_{2}\left(R\right) \end{array}$$

We $\text{set}V_{0}≐V\left(0\right)$ , and let


(33) $$\left\{\begin{array}{l} {\bar{G}}_{1}≐\overset{r_{out}}{\underset{r_{in}}{\int }}g_{1}\left(s\right)ds\\ {\bar{G}}_{2}≐\overset{R}{\underset{r_{in}}{\int }}g_{2}\left(s\right)ds \end{array}\right.$$

There is the maximum collision avoidance potential function $G_{1}^{\max}=k{\bar{G}}_{1}$ and the maximum collision avoidance potential function $G_{2}^{\max}=k{\bar{G}}_{2}$ . Then, Eq. (32) can be transformed as


(34) $$\{\begin{array}{l} V_{0}\leq k{\bar{G}}_{1}\\ V_{0}\leq k{\bar{G}}_{2} \end{array}$$

To ensure collision avoidance, connectivity preservation, and multi-subgroup formation generation, it is necessary to set k to the larger value between $\frac{V_{0}}{G_{1}}$ and $\frac{V_{0}}{G_{2}}$ . Let $\Gamma =\left\{\left(\bar{x}\left(t\right),v\left(t\right)\right)|V\left(t\right)\leq V\left(0\right),\forall t\geq 0\right\}$ . According to LaSalle’s invariance principle (Tong et al., 2015), each of the solutions starting in Γ closes to the largest invariant set $W=\left\{\left(\bar{x}\left(t\right)\text{,}v\left(t\right)\right)\in \Gamma |\dot{V}\left(t\right)=0\right\}$ as t→∞ . In Eq. (30), $\dot{V}=0$ if ${\hat{v}}_{i}=0,\forall i\in V$ which yields $\dot{V}=0$ and $v_{1}=v_{2}=\cdots =v_{n}=0$ ; i.e., this indicates that all agents have reached the same speed and all have a speed of 0. In other words, the speed of the agent reaches a stable consensus. When $t\to \infty ,u_{i}$ can be simplified from Eq. (20) to:


(35) $$\begin{array}{rr} u_{i}={\dot{v}}_{i}= & -\omega_{i}^{2}\underset{j\in N_{i}^{q}}{\sum }\nabla_{x_{i}}G_{1}\left(x_{ij}\right)-\omega_{i}^{2}\underset{j\in N_{i}^{l}}{\sum }\nabla_{x_{i}}G_{2}\left(x_{ij}\right)\\ & -\alpha \underset{j\in N_{i}}{\sum }S\left(\varepsilon_{i}^{\rho },\varepsilon_{j}^{\rho },v_{m}\right)=0 \end{array}$$

When the system converges, $x_{ij}=\rho_{ij},u_{i}$ can be rewritten as


(36) $$u_{i}=-\alpha \underset{j\in N_{i}}{\sum }S\left(\varepsilon_{i}^{\rho },\varepsilon_{j}^{\rho },v_{m}\right)=-\underset{j\in N_{i}}{\sum }\alpha \left(\varepsilon_{i}^{\rho }-\frac{\omega_{i}}{\omega_{j}}\varepsilon_{j}^{\rho }\right)=0$$

accordingly,


(37) $$u=-\alpha \hat{L}\bar{x}=-\alpha \Lambda L\hat{x}=0_{n}$$

We expect that the control input $u_{i}$ to be 0 only when the agents in MAS reach multi-consensus.

The above proof is in the case where the MAS connection is a static network. We need to extend Lyapunov’s stability proof to the case where the network topology is dynamic. We define the network topology graph at moment t as $G\left(t\right).L_{G\left(t\right)}$ is the Laplace matrix at moment t . The $\omega_{i}$ changes when the subgroup assigned by the agent changes dynamically (Olfati-Saber, 2006). In line with this, the multi-consensus control method is a segmented continuous function. The control input function jumps when the network topology graph of the target formation changes. From Choi et al. (2019), we can obtain that the system whose network topology changes with time is the switching system.

Then, this study requires a stability analysis of the above system with dynamic networks. In this study, we refer to the definition of residence time for analysis. In the switching system, this study refers to the concept of minimum dwell time (MDT). In the concept, the subsystem before and after switching is a progressively stable system, and then, the switching system is also a progressively stable system (Xu et al., 2021). We assume that the network changes at moment $t_{k}$ and the next moment of change is $t_{k+1}$ . However, the topology is fixed in the time from $t_{k}$ to $t_{k+1}$ and this time interval is large enough. The residence time is defined as $\tau \left(t\right)$ in this study and refers to the time interval between moments of network change, as follows:


(38) $$\tau \left(t_{k}\right)=t_{k+1}-t_{k},\forall k\in N$$

**Assumption 1.** *In this study, the minimum residence time is defined as* $\tau_{d}$ *, and the minimum residence time* $\tau_{d}$ *is large enough to ensure the switching system stability. All time intervals of network topology switching are longer than the minimum residence time, that is,* $\tau \left(t_{k}\right)\geq \tau_{d}>0$ *. Following this, the ensemble of all possible network topology diagrams in the MAS system is* $G_{c}$ .

Furthermore, according to Theorem 1, if the initial topological map of the MAS is an element in $G_{c}$ , then the transformed topological map remains in the $G_{c}$ under the action of the control methods, i.e., if $G_{0}\in G_{c}$ , then $G_{t}\in G_{c},\forall t\geq 0$ .

**Corollary 1.** *If Theorem 1 and Assumption 1 established, then this MAS can have stability in dynamic network.*

***Proof.*** Since $\tau_{d}\leq \tau \left(t_{k}\right)$ , the network topology of the MAS is asymptotically stable from $G_{0}$ . The system network topology $G_{t}$ at time t is asymptotically stable, and then, the intermediate switching system is also stable.

It is worth noting that the control method (Eq. 20) proposed in this study requires a network constraint that is the initial network connection of the MAS. The algorithm in this study allows networks between subgroups to be disconnected. Although the agents may not receive the information of other agents in the same sub-term, each subgroup can reach the split formation based on ω .

## 4 Simulation

### 4.1 Multi-consensus formation simulation

This part is the simulation part of the study, which can verify our proposed algorithm. This study generates three formations using 20 agents with initial network connections. The initial position of all agents is random within a certain range. The number of agents for the three separate formations is 8, 6, and 6, respectively (Tong et al., 2015). They have slightly different degrees of intelligence ( ω ), 1, 1.1, and 1.2.

The whole system satisfies the initial network constraints. The initial network is connected between all agents. In addition, the algorithm allows the agents between each grouping to exist disconnected at the beginning. The formation configuration that the simulation wants to form in this section is a diamond, rectangle, and triangle. Table 1 contains the relevant parameters required for the simulation in this subsection. The state space of agents is two-dimensional in the simulation.

**Table 1:** Parameters for numerical simulations.

Parameter	Value
Communication boundary, R	50[m]
Formation-limit boundary, $R^{\prime }$	40[m]
Exclusion outer boundary, $r_{out}$	5[m]
Exclusion inner boundary, $r_{in}$	0.5[m]
Potential function gain, ( $k_{1},k_{2}$ )	(10, 5)
Control parameters, ( α,β )	(1.8, 1.8)
Intelligence degree, ω	(1, 1.1, 1.2)
Communication attenuation rate, τ	0.6
Connectivity lower bound, ϵ	0.2

**Figure 4:**
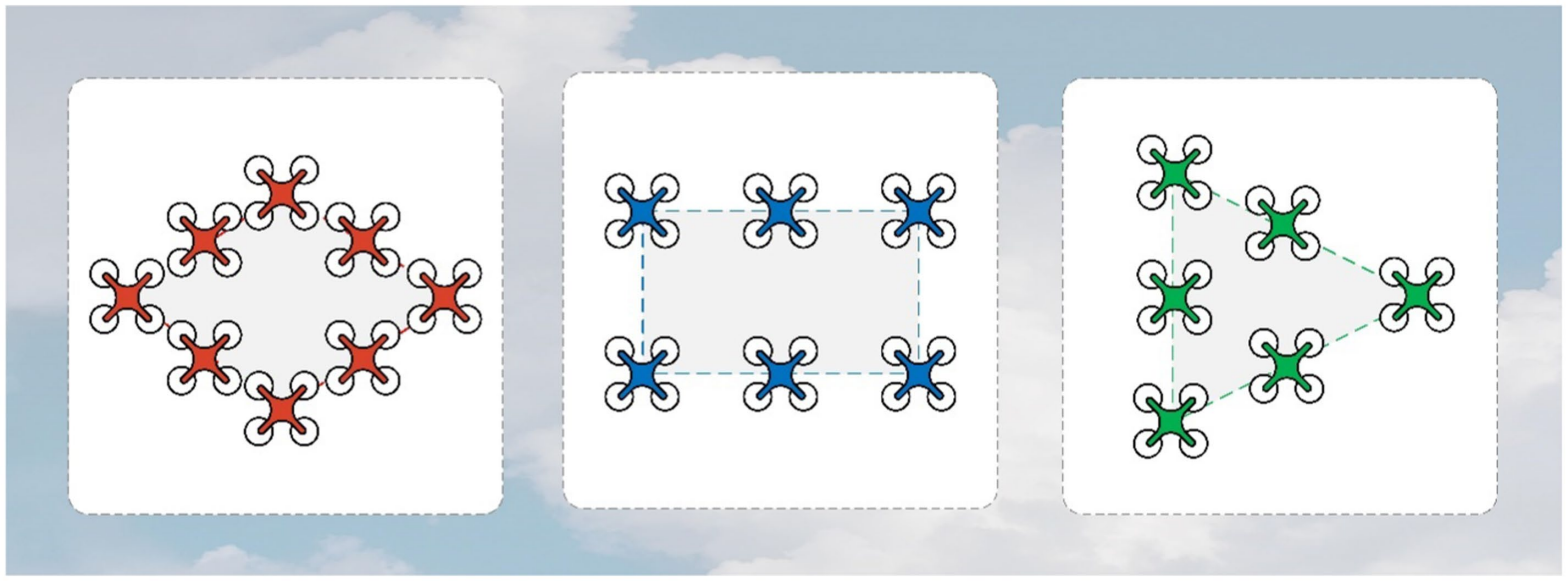
Desired formation for the simulation.

**Figure 5:**
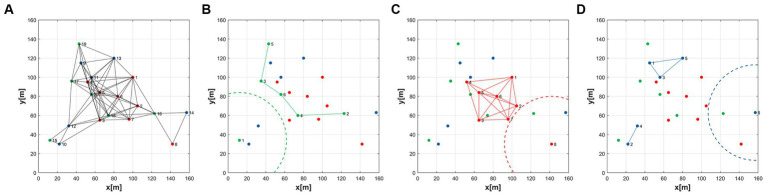
Initial network topology configuration of the MAS. **(A)** Initial agent distribution, **(B)** Green group(ω = 1), **(C)** Red group(ω = 1.1), **(D)** Blue group(ω = 1.2).

The details of the network connections in this simulation are shown in Figure 4. In Figure 5A, the black line indicates that the distance between the agents is less than R ; that is, there is connectivity between them. The entire MAS network is initially connected. In Figure 5B, it shows the network connections between the green subgroups. Similarly, the connections between the red and blue subgroups are indicated in Figures 5C,D, respectively. The dotted line refers to the circle with the agent as the center and the communication range R as the radius.

**Figure 6:**
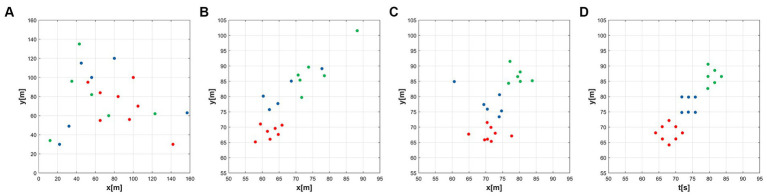
Simulation results of the multi-subgroup formation control. **(A)** t = 0s, **(B)** t = 2s, **(C)** t = 5s, **(D)** t = 10s.

Through Figures 6A–D, the temporal process of simulation is displayed, revealing the formation process of the formations. Over time, the agents gradually adjust their positions and velocities to achieve the desired formation shapes.

**Figure 7:**
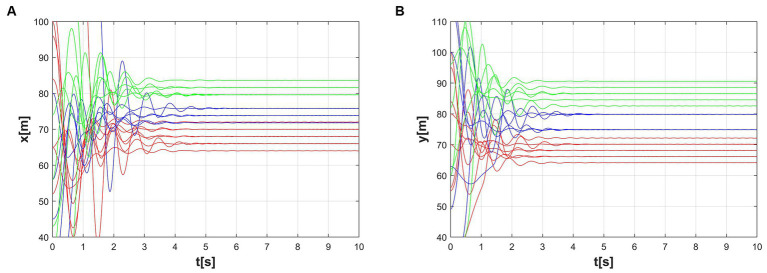
Consensus of two different latitudes. **(A)** The first dynamic multi-consensus, **(B)** The second dynamic multi-consensus.

The first and the second-dimensional consensus among multiple agents are shown in Figures 7A,B. By observing the graphs and data, we can evaluate the performance of the algorithm and its adaptability to different formation shapes. As seen in the figures, the formation process is smooth, consistency is quickly achieved, and the communication connections between agents remain stable. This indicates that the algorithm has good feasibility in practical applications.

### 4.2 Dynamic formations

This subsection is about the simulation of the dynamic network of the MAS. It shows the flexibility of the algorithm in this study we proposed.

**Figure 8:**
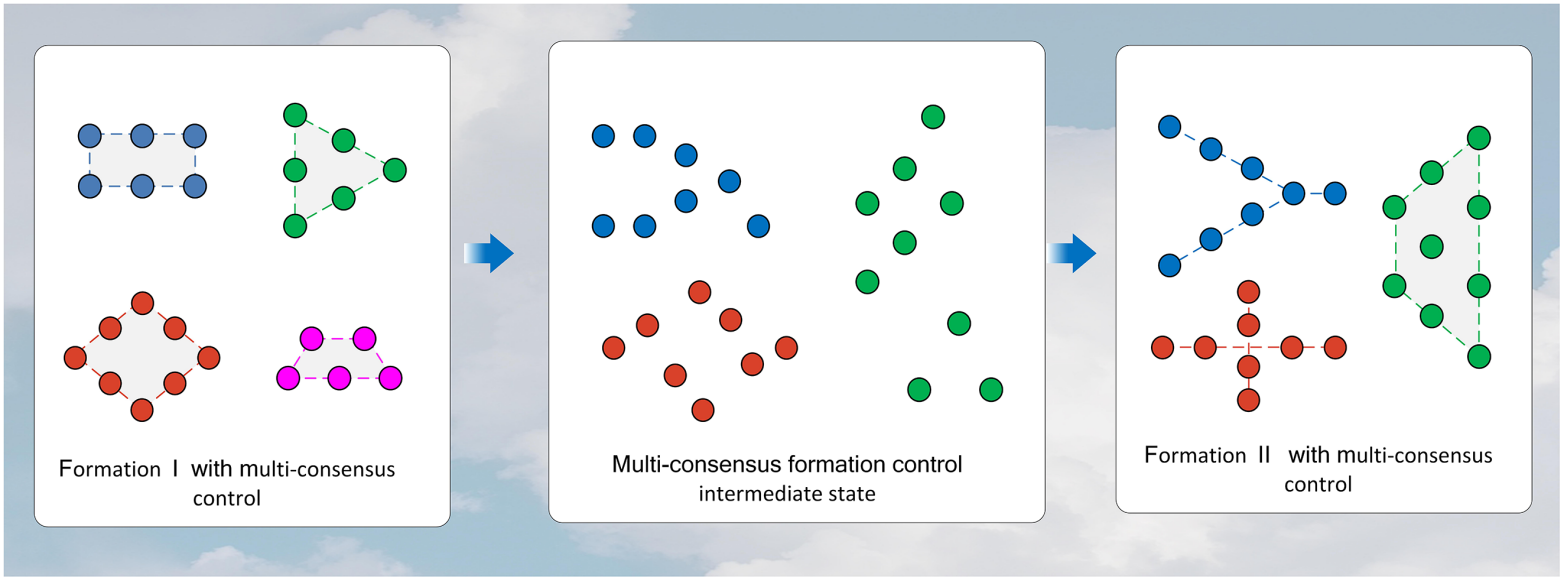
Scenario of time-varying formations.

The simulation in this subsection increases the number of agents to 25. In the simulation process of this section, the first graph of Figure 8 is formed from the disordered state, and then, the second graph is formed. On the one hand, we changed the number of agents, and on the other hand, we changed the configuration of the formed formations. The initial state of all agents is random within a certain range. The other parameter domains used for the simulation are the same as in Table 1.

**Figure 9:**
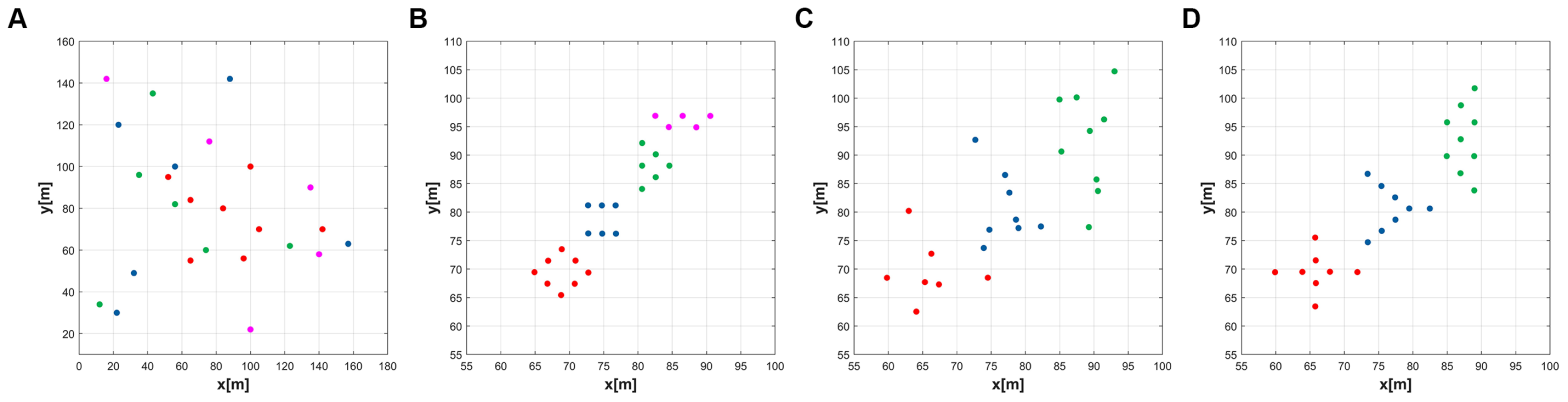
Formation simulation history process in the time-varying scenario. **(A)** t = 0s, **(B)** t = 10s, **(C)** t = 12s, **(D)** t = 20s.

**Figure 10:**
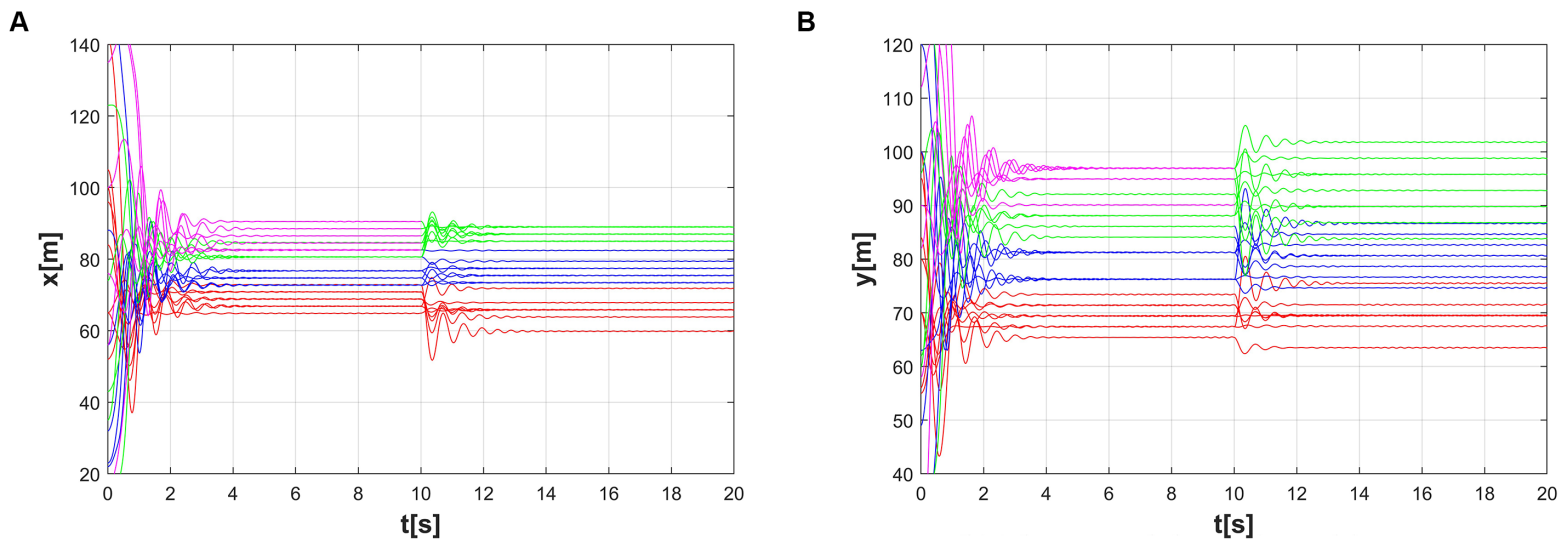
Multi-consensus formation during the time-varying scenario. **(A)** The first dynamic multi-consensus, **(B)** The second dynamic multi-consensus.

Figures 9A–D refers to the time course of the dynamic formation simulation. Although the formation of the targets is changing dynamically, the system establishes an ordered configuration under the action of the control method. The first multi-consensus and second multi-consensus between multiple agents are shown in Figures 10A,B.

## 5 Conclusion

In this study, we designed an improved APF method for distributed multi-formation control that enables multi-formation tasks and places restrictions on the inputs, which makes the system secure. A distributed controller is designed based on an improved APF method that ensures collision avoidance and maintains the communication topology during formation changes. Simulation results demonstrate the effectiveness of our designed controller. In future work, the optimization of multi-intelligence assignments in multiple formations can be considered. The allocation problem requires consideration of the environment and task requirements and can be considered to be solved by optimizing the allocation algorithm.

## Data availability statement

The original contributions presented in the study are included in the article/supplementary material, further inquiries can be directed to the corresponding author.

## Author contributions

XC: Conceptualization, Writing – original draft, Writing – review & editing. JJ: Conceptualization, Methodology, Validation, Writing – original draft, Writing – review & editing. YL: Methodology, Writing – original draft, Writing – review & editing. BH: Conceptualization, Writing – original draft, Writing – review & editing.
